# Low oxygen tension increased fibronectin fragment induced catabolic activities - response prevented with biomechanical signals

**DOI:** 10.1186/ar4346

**Published:** 2013-10-25

**Authors:** Eleanor Parker, Sandrine Vessillier, Belinda Pingguan-Murphy, Wan Abu Baker Wan Abas, Dan L Bader, Tina T Chowdhury

**Affiliations:** 1 Institute of Bioengineering, School of Engineering and Materials Science, Queen Mary University of London, Mile End Road, London E1 4NS, UK; 2 National Institute for Biological Standards and Control, Biotherapeutics Group, South Mimms, Potters Bar, Hertfordshire EN6 3QG, UK; 3 Department of Biomedical Engineering, Faculty of Engineering, University of Malaya, 50603, Kuala Lumpur, Malaysia

## Abstract

**Introduction:**

The inherent low oxygen tension in normal cartilage has implications on inflammatory conditions associated with osteoarthritis (OA). Biomechanical signals will additionally contribute to changes in tissue remodelling and influence the inflammatory response. In this study, we investigated the combined effects of oxygen tension and fibronectin fragment (FN-f) on the inflammatory response of chondrocytes subjected to biomechanical signals.

**Methods:**

Chondrocytes were cultured under free-swelling conditions at 1%, 5% and 21% oxygen tension or subjected to dynamic compression in an *ex vivo* 3D/bioreactor model with 29 kDa FN-f, interleukin-1beta (IL-1β) and/or the nitric oxide synthase (NOS) inhibitor for 6 and 48 hours. Markers for catabolic activity (NO, PGE_2_), tissue remodelling (GAG, MMPs) and cytokines (IL-1β, IL-6 and TNFα) were quantified by biochemical assay. Aggrecan, collagen type II, iNOS and COX-2 gene expression were examined by real-time quantitative PCR. Two-way ANOVA and a *post hoc* Bonferroni-corrected *t*-test were used to analyse data.

**Results:**

Both FN-fs and IL-1β increased NO, PGE_2_ and MMP production (all *P* < 0.001). FN-f was more active than IL-1β with greater levels of NO observed at 5% than 1% or 21% oxygen tension (*P* < 0.001). Whilst FN-f reduced GAG synthesis at all oxygen tension, the effect of IL-1β was significant at 1% oxygen tension. In unstrained constructs, treatment with FN-f or IL-1β increased iNOS and COX-2 expression and reduced aggrecan and collagen type II (all *P* < 0.001). In unstrained constructs, FN-f was more effective than IL-1β at 5% oxygen tension and increased production of NO, PGE_2_, MMP, IL-1β, IL-6 and TNFα. At 5% and 21% oxygen tension, co-stimulation with compression and the NOS inhibitor abolished fragment or cytokine-induced catabolic activities and restored anabolic response.

**Conclusions:**

The present findings revealed that FN-fs are more potent than IL-1β in exerting catabolic effects dependent on oxygen tension via iNOS and COX-2 upregulation. Stimulation with biomechanical signals abolished catabolic activities in an oxygen-independent manner and NOS inhibitors supported loading-induced recovery resulting in reparative activities. Future investigations will utilize the *ex vivo* model as a tool to identify key targets and therapeutics for OA treatments.

## Introduction

Animal and *in vitro* studies have provided convincing evidence for a role of matrix degradation products in regulating cartilage homeostasis and driving osteoarthritis (OA) disease progression [1-3]. In chondrocytes, fragments derived from fibronectin initiate both catabolic and anabolic signalling cascades in a concentration-dependent manner [3,4]. At low concentration, fragments augment anabolic processes and facilitate reparative processes when the extracellular matrix is damaged. However, if fragment levels increase above a certain threshold, the pathways switch from anabolic to catabolic and accelerate matrix damage mediated by production of matrix metalloproteinases (MMPs) and cytokines [2]. The importance of fragment-induced damaging effects were highlighted in previous clinical studies, which reported elevated levels of fibronectin fragments (FN-fs) in osteoarthritic or rheumatoid cartilage and OA synovial fluids [5-8]. The catabolic environment up-regulates tissue remodelling but the response will be influenced by mechanical factors which interfere with the pathways [9,10]. The mediators that initiate the early phase of matrix damage are therefore complex and involve both mechanical and biological factors. In addition, the way in which biomechanical signals modulate fragment-induced mechanisms for repair and/or degradation in early stage OA are unclear and require further investigation.

Indeed, the amino-terminal FN-f has been shown to have potent catabolic activities leading to enhanced levels of nitric oxide (NO), prostaglandin E_2_ (PGE_2_) and MMPs in human or bovine cells cultured in 3D agarose, monolayer or explant models [1,3,11-13]. The signalling pathways involve the mitogen activated protein kinase (MAPK) and nuclear factor-kappa B (NFκB) cascades mediated by stimulation of integrin receptors, leading to suppression of proteoglycan synthesis and increased proteoglycan depletion [14,15]. Furthermore, inducible nitric oxide synthase (iNOS) inhibitors have been shown to reduce the catabolic effect in cartilage explants treated with FN-f and repair damaged tissue by facilitating anabolic processes [12]. Recently, we showed that intermittent compression applied in a dynamic manner inhibits FN-f induced NO and PGE_2_ production and restores matrix synthesis in chondrocytes cultured in agarose constructs [16]. In this study, treatment with iNOS inhibitors and stimulation with mechanical signals was shown to prevent FN-f-induced catabolic response. In addition, fibronectin concentrations were demonstrated to increase by cyclic impact load and alter matrix synthesis in cartilage explants [17]. Mechanical loading conditions that mimic injury and overloading may accelerate mild damage with an early rebuilding phase by increasing MMPs, matrix fragment levels and metabolic activity [18]. However, the response will at least, in part, be dependent on the type of mechanical loading regime, its duration and whether loading was applied during the early or late stage of the disease process. It is, therefore, plausible that physiological mechanical signals compete with the catabolic pathways induced by the matrix fragments and contribute to early reparative signals.

Furthermore, the oxygen tension of cartilage will influence the response of chondrocytes to inflammatory factors and biomechanical signals. In OA, the tissue is more hypoxic than normal cartilage with pathophysiological levels less than 5% leading to increased production of NO and PGE_2_ release in tissues involving the cartilage and meniscus [19-21]. The interactions of inflammatory mediators, such as interleukin-1β (IL-1β), with oxygen tension has detrimental effects on matrix turnover, which, in turn, affects the ability of the cells to respond to mechanical loading, possibly through the disruption of normal integrin-based signals [19-21]. Given the potential inflammatory effects of hypoxia on cell metabolism, it is highly likely that oxygen tension will affect the response of chondrocytes to both matrix fragments and mechanical stimuli. However, to date, no research groups have examined the combined effect of fragments, oxygen tension and biomechanical signals in chondrocytes. The present study, therefore, investigated the effects of oxygen tension and FN-f on catabolic and anabolic activities in chondrocyte/agarose constructs subjected to dynamic compression and compared the response to constructs treated with IL-1β.

## Methods

### Chondrocyte isolation and culture in agarose constructs

This study involves bovine cells procured from a local abbatoir with authorization from the relevant meat inspectors (Dawn Cardington, Bedfordshire, UK). It does not involve humans, human tissue or experimentation on animals. Cartilage explants were obtained from the metacarpalphalangeal joints of 18-month-old cattle and diced, as previously described [22,23]. The tissue was incubated on rollers for 1 h at 37°C in Dulbecco’s Modified Eagle’s Medium supplemented with 20% (v/v) foetal calf serum (DMEM + 20% FCS), 2 μM L-glutamine, 5 μg/ml penicillin, 5 μg/ml streptomycin, 20 mM Hepes buffer, and 0.85 μM L-ascorbic acid in addition to 700 unit/ml pronase, and for a further 16 h at 37°C in medium supplemented with 100 unit/ml collagenase type XI (All from Sigma-Aldrich Ltd, Gillingham, Dorset, UK). The chondrocyte suspension was washed and the cells counted using a haemocytometer. Cell viability was calculated with the trypan blue exclusion assay and resuspended in medium at a concentration of 8 × 10^6^ cells/ml. The cell suspension was added to an equal volume of molten 6% (w/v) agarose type VII in Earle’s Balanced Salt Solutions (EBSS) to yield a final cell concentration of 4 × 10^6^ cells/ml in 3% (w/v) agarose (Sigma-Aldrich Ltd, Gillingham, Dorset, UK). The cell/agarose suspension was transferred into a sterile stainless steel mould, containing holes, 5 mm in diameter and 5 mm in height, and allowed to gel at 4°C for 10 minutes. Constructs were transferred into wells of a 24-well culture plate and immediately subjected to *ex vivo* conditions.

### Effects of oxygen tension and FN-f in chondrocyte/agarose constructs

**Figure 1:**
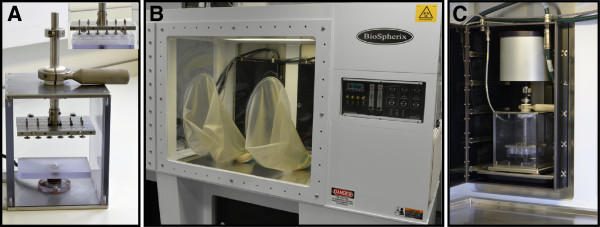
**The *ex vivo *system.** Bose bioreactor **(A)** integrated with the Biospherix **(B)** to facilitate uninterrupted *ex vivo* conditions, under controlled oxygen tension within the incubator **(C)**. The inset in **(A)** shows custom designed compressive mounting plate with loading pins positioned above a 24-well culture plate.

**Figure 2:**
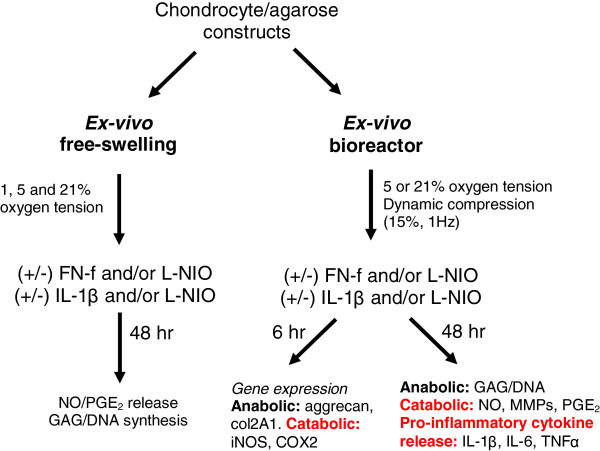
**Summary of the *ex vivo *test conditions and types of analysis.** Chondrocyte/agarose constructs were cultured *ex vivo* under free-swelling conditions (1, 5 and 21%) or subjected to dynamic compression (15%, 1 Hz) at 5 and 21% oxygen tension. At the end of the experiment, constructs and/or media samples were analysed by biochemical assay or RNA was extracted from constructs, reverse transcribed and gene expression quantified by RT-qPCR.

The effects of 1, 5 and 21% oxygen tension were examined in constructs cultured under free-swelling conditions in a glove-box style workspace integrated with the Biospherix Ltd, Lacona, NY, USA incubator to ensure that the experimental conditions during set-up were uninterrupted (Figure 1). 1 ml DMEM + 20% FCS was supplemented with 0 or 1 μm of 29 kDa NH_2_-heparin-binding FN-f (generous gift from Prof Gene Homandberg) and/or 1 mM of L-*N*-(1-iminoethyl)-ornithine (L-NIO). This agent inhibits all isoforms of the nitric oxide synthase enzymes (Merck Chemicals, Nottingham, UK) for 48 hr. FN-fs were isolated from cathepsin D and thrombin digests of fibronectin from plasma adsorption, as previously described [24]. In addition, the FN-f-induced response was compared to constructs treated with 0 or 10 ng/ml IL-1β (Peprotech EC Ltd, London, UK) and/or L-NIO at 1, 5 and 21% oxygen tension. The *ex vivo* conditions are summarized in Figure 2.

### Application of dynamic compression

In separate experiments, a novel *ex vivo* bioreactor (Bose ElectroForce, Gillingham, UK) was used to apply dynamic compression to constructs cultured at 5 or 21% oxygen tension (Figure 1). Constructs were transferred into individual wells of a 24-well culture plate (Figure 1A, inset) and mounted within the bioreactor device that was integrated with the Biospherix incubator (Figure 1B, C). The medium was supplemented with either 0 or 1 μM FN-f or 10 ng/ml IL-1β and/or 1 mM L-NIO and the experimental conditions during set-up were uninterrupted. Constructs were subjected to intermittent compression under unconfined conditions, with a profile of 10 minutes compression followed by a 5- hour 50-minute unstrained period for both the 6- and 48-hour culture periods, as previously described [16,25]. The compression regime was applied in a dynamic manner with strain amplitude of 0 to 15% in a sinusoidal waveform at a frequency of 1 Hz and resulted in duty cycles which ranged from 600 to 4,800 cycles. Control constructs were unstrained, but were maintained within the *ex vivo* bioreactor. The *ex vivo* conditions are summarized in Figure 2 and the 48-hour time point was found to be optimal when measuring production of inflammatory mediators and GAG synthesis.

### Biochemical analysis

At the end of the 48-hour experiment, the constructs and corresponding media were removed and stored at -20°C prior to analysis. Constructs were digested overnight with 2.8 unit.ml^-1^ papain and 10 unit/ml agarase at 37°C (both Sigma-Aldrich Ltd, Gillingham, Dorset, UK), as previously described [22,26]. Media samples were analysed for total MMP activity using a fluorogenic substrate assay. A 20-μl sample was mixed with 10 μM Dnp-PChaGCHAK(Nma) fluorogenic MMP substrate in 50 μl buffer (50 mM HEPES, 10 mM CaCl_2_, 10 μM ZnCl_2_, 0.05% Brij-35, pH 7.0) in each well of a 96-well plate (Enzo Life Sciences, Exeter, UK). Reactions were measured by fluorescence at excitation and emission values of 340 and 440 nm, respectively. TNF-α (Bethyl Laboratories Inc., Montgomery, AL, USA), IL-1β and IL-6 (both from Thermo Scientific, Cramlington, Northumberland, UK) were quantified by ELISA according to manufacturers’ instructions. Absolute concentrations of nitrite (μM), a stable end-product of NO, were measured in the culture media using a spectrophotometric method based on the Griess assay. PGE_2_ production was measured in the culture media by EIA (R&D Systems, Abingdon, Oxfordshire, UK). Total DNA was determined from agarase/papain digest using the Hoescht 33258 method. GAG synthesis was measured in constructs and media samples by DMB assay. These methods have been previously detailed [22,26].

### RNA isolation, reverse transcription and real-time quantitative PCR

At the end of the six-hour bioreactor experiment, RNA was isolated from chondrocytes cultured in agarose using the QIAquick Spin gel extraction and RNeasy kit (Qiagen, Crawley, West Sussex, UK), as previously described [27]. The six-hour time point was found to be optimal when examining gene expression [25]. DNA-free DNase treatment and removal reagents were used to eliminate any contaminating DNA from the RNA sample (Applied Biosystems, Warrington, UK). RNA was quantified on the Nanodrop ND-1000 spectrophotometer (LabTech, Uckfield, East Sussex, UK), and reverse transcription was performed with Enhanced Avian RT First Strand cDNA synthesis kit, oligo(dT)23 primer, and a total of 200 ng of RNA (Sigma-Aldrich Ltd, Gillingham, Dorset, UK). Real-time quantitative polymerase chain (qPCR) assays coupled with Molecular Beacons were performed in 25-μl reaction mixtures containing 1 μl cDNA, 12.5 μl JumpStart Taq PCR Master Mix, primer pairs/probes with concentrations detailed in Table 1 and nuclease-free PCR-grade water to 25 μl (Sigma Genosys, Cambridge, UK). Each sample was run in duplicate on the 96-well thermal system of the Mx3000P quantitative PCR instrument (Stratagene, Amsterdam, Netherlands). Thermocycling conditions comprised an initial polymerase activation step at 95°C for 10 minutes, followed by denaturation of 35 cycles at 95°C for 30 seconds, annealing at 55°C for 1 minute, and extension at 72°C for 1 minute. The real-time PCR efficiencies (E) of amplification for each target were defined according to the relation, E = 10(-1/slope) and revealed efficiency values ranging from 1.96 to 2.05.

**Table 1:** Beacon designer sequences and real-time reaction efficiencies of qPCR assays

**Gene**	**Accession number**	**Sequences**	**nM**	**Efficiency**
iNOS	U14640	**Probe:5′-FAM-CGCGATC** **CCTGCTTGGTGGCGAAGATGAGC** **GATCGCG-**	200	
		**DABCYL-3′**	200	2.01
		**Forward: 5′-GTAACAAAGGAGATAGAAACAACAGG-3′**	200	(± 0.08)
		**Reverse: 5′-CAGCTCCGGGCGTCAAAG-3′**		
		**Probe: 5′-FAM-**		
COX-2	AF031698	**CGCGATC** **GTCAGAAATTCGGGTGTGGTACAGTT** **GATCGCG-DABCYL-3′**	200	
		**Forward: 5′-CGAGGTGTATGTATGAGTGTAGG-3′**	300	1.99
		**Reverse: 5′-GTTGGGAGTGGGTTTCAGG-3′=**	300	(± 0.04)
		**Probe: 5′-FAM-**	200	
Aggrecan	U76615	**CGCGATC** **CACTCAGCGAGTTGTCAGGTTCTGA** **GATCGCG-DABCYL-3′**		2.05
		**Forward: 5′-TGGTGTTTGTGACTCTGAGG-3′**	100	(± 0.07)
		**Reverse: 5′-GATGAAGTAGCAGGGGATGG-3′**	200	
Collagen type II	X02420	**Probe: 5′-FAM-CGCGAT** **GCGTCAGGTCAGGTCAGCCAT** **ATCGCG-**		
		**DABCYL-3′**	200	
		**Forward: 5′-AAACCCGAACCCAGAACC-3′**	100	1.96
		**Reverse: 5′-AAGTCCGAACTGTGAGAGG-3′**	100	(± 0.08)
GAPDH	U85042	**Probe: 5′-HEX-CGCGATC** **CACCATCTTCCAGGAGCGAGATCC** **GATCGCG-**		
		**DABCYL-3′**	200	1.96
		**Forward: 5′-TTCAACGGCACAGTCAAGG-3′**	200	(± 0.01)
		**Reverse: 5′-TTCAACGGCACAGTCAAGG-3′**	200	

Note: Real-time reaction efficiencies of qPCR assays with optimal primer and probe concentrations are shown. Probes contain FAM or HEX as a 5′- reporter dye and DABCYL as 3′- quencher with the arm sequences underlined.

Fluorescence data were collected during the annealing stage of amplification, and data were analyzed on the MxPro qPCR software (version 3, Agilent Technologies Inc, Wokingham, Berkshire, UK). Baselines and thresholds were automatically set by the RG-3000 qPCR software and used after manual inspection. The cycle threshold (Ct) value for each duplicate reaction was expressed as the mean value, and the results were exported into Microsoft Excel for further analysis. Relative quantification of collagen type II, aggrecan, COX-2 and iNOS signals were estimated by normalizing each target to the reference gene, GAPDH, and to the calibrator sample (untreated, unstrained condition) by a comparative Ct approach. For each sample, the ratio of target ∆Ct and reference ∆Ct was calculated, as previously described [27].

### Statistics

For free-swelling studies, data represent the mean and SEM values of 12 replicates from three separate experiments. For the *ex vivo*/bioreactor experiments, biochemical and gene-expression data represent the mean and SEM values of up to 12 replicates from at least three separate experiments. Statistical analysis was performed with a two-way analysis of variance (ANOVA) and the multiple *post hoc* Bonferroni-corrected *t* tests to compare differences between the various treatment groups, as indicated in the figure legend. For gene-expression data, ratio values were log transformed before analysis by a two-way ANOVA and a *post hoc* Bonferroni-corrected *t* test. In all cases, a level of 5% was considered statistically significant (*P* <0.05).

## Results

### FN-f maximally increased catabolic activities at 5% oxygen tension

**Figure 3:**
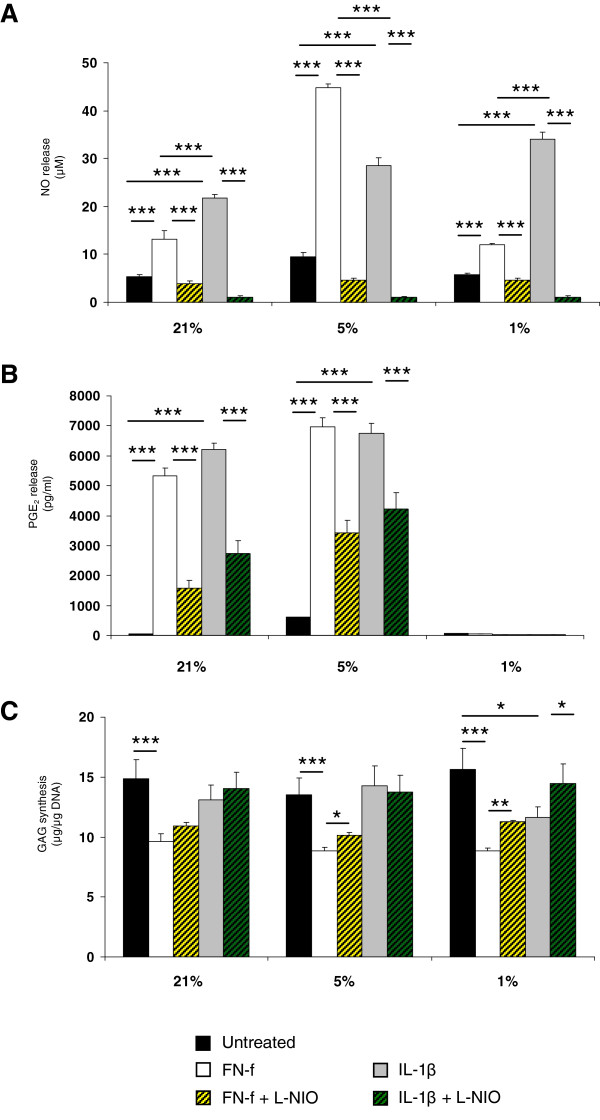
**Effect of oxygen tension and FN-f on catabolic and anabolic activities.** Chondrocyte/agarose constructs were cultured under uninterrupted experimental conditions with 0 or 1 μM FN-f or 10 ng/ml IL-1β in the presence or absence of 1 mM L-NIO at 1, 5 and 21% oxygen tension for 48 hours, where NO release **(A)**, PGE_2_ rowsep="1"production **(B)** rowsep="1"and GAG synthesis **(C)**. Error bars represent the mean and SEM of 12 values from three separate experiments.

The ability of FN-f to influence the levels of NO, PGE_2_ and GAG synthesis in an oxygen-dependent manner were compared to IL-1β in constructs cultured for 48 hours (Figure 3). At 1, 5 and 21% oxygen tension, NO production was enhanced with FN-f or IL-1β compared to untreated controls (all *P* <0.001; Figure 3A). At 5% oxygen tension, FN-f increased NO production (45 μM) with values significantly greater than cytokine-treated (28 μM) constructs (*P* <0.001). At all oxygen tensions, co-incubation with L-NIO reduced fragment or cytokine-induced NO levels with values returning to basal levels (<5 μM). PGE_2_ release was increased with either FN-f or IL-1β at 5 and 21% oxygen tension (*P* <0.001) but not at 1% oxygen tension (Figure 3B). Co-incubation with L-NIO partially reduced PGE_2_ levels in FN-f treated constructs cultured at 5 and 21% oxygen tension. Furthermore, FN-f reduced GAG synthesis at 1, 5 and 21% oxygen tension (*P* <0.001; Figure 3C). In contrast, the cytokine decreased GAG synthesis at 1% oxygen tension (*P* <0.01; Figure 3F) but not at 5 or 21% oxygen tension. At 1 or 5% oxygen tension, fragment or cytokine-induced inhibition on GAG synthesis was reversed with L-NIO (both *P* <0.05).

### Compression abolished FN-f induced catabolic effects at 5 and 21% oxygen tension

**Figure 4:**
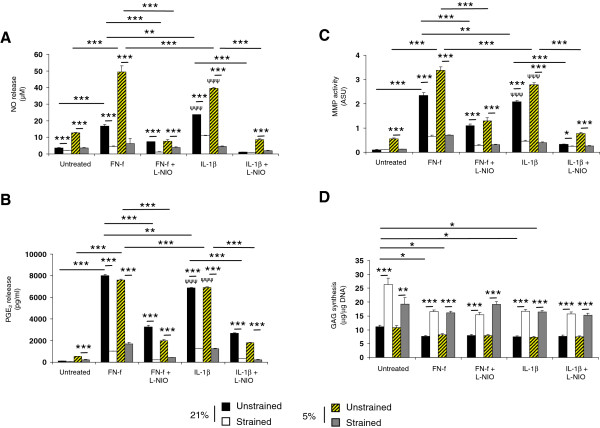
**Effect of oxygen tension, FN-f and dynamic compression on catabolic and anabolic activities. ** Chondrocyte/agarose constructs were cultured at 5 and 21% oxygen tension with 0 or 1 μM FN-f or 10 ng/ml IL-1β and/or 1 mM L-NIO for 48 hour, where NO release **(A)**, PGE_2_ production **(B)**, MMP activity **(C)** and GAG synthesis **(D)**. Error bars represent the mean and SEM of 8 to 12 replicates from three separate experiments, where (*) indicates comparisons between the different treatment groups and (ψ) indicates comparisons in unstrained constructs between untreated and IL-1β at 5 or 21% oxygen tension. All other comparisons were not significant (not indicated).

Since the catabolic effects in response to FN-f were maximal at 5% oxygen tension, further *ex vivo* studies examined the effect of dynamic compression in constructs cultured at 5 and 21% oxygen tension and compared the response to IL-1β. Figure 4 reveals that dynamic compression inhibits NO release at 5 and 21% oxygen tension (*P* <0.001; Figure 4A). In unstrained constructs, FN-f enhanced NO production (*P* <0.001) with values highest at 5% oxygen tension when compared to the effects of IL-1β (*P* <0.001). The enhanced production of NO by fragment, cytokine or low oxygen tension was reduced with dynamic compression (all *P* <0.001; Figure 4A). Stimulation with compression and the NOS inhibitor reduced NO levels further in fragment or cytokine treated constructs (all *P* <0.001; Figure 4A).

At 5% oxygen tension, dynamic compression inhibits PGE_2_ release in untreated constructs (*P* <0.001; Figure 4B). In unstrained constructs, the presence of the fragment or cytokine enhanced PGE_2_ release with values broadly similar and ranging from 7.6 to 8.0 pg/ml for constructs cultured at 5 or 21% oxygen tension. Stimulation with dynamic compression and/or L-NIO reduced PGE_2_ levels to basal values in an oxygen-independent manner (all *P* <0.001) with the magnitude in inhibition ranging between 77 and 87%.

In untreated samples, total MMP activity was inhibited with dynamic compression at 5% oxygen tension. The fragment or cytokine increased MMP activity with maximal values at 5% oxygen tension for constructs cultured with FN-f when compared to IL-1β (*P* <0.001). At 5 or 21% oxygen tension, stimulation with dynamic compression or the NOS inhibitor abolished fragment or cytokine induced MMP activity (all *P* <0.001; Figure 4C). In addition, dynamic compression increased GAG synthesis (*P* <0.001; Figure 4D) with a greater magnitude in stimulation for constructs cultured at 21% oxygen tension (136%) when compared to 5% (79%). At 5 and 21% oxygen tension, the presence of the FN-f or IL-1β reduced GAG synthesis (all *P* <0.05; Figure 4D). This response was reversed by stimulation with dynamic compression and L-NIO at 5 and 21% oxygen tension.

### Compression inhibits FN-f induced gene expression at 5 and 21% oxygen tension

**Figure 5:**
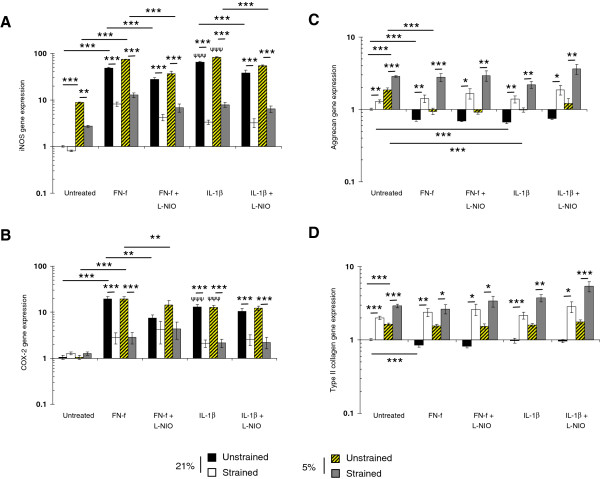
**Effect of oxygen tension, FN-f and dynamic compression on gene expression. ** Chondrocyte/agarose constructs were cultured at 5 and 21% oxygen tension with 0 or 1 μM FN-f or 10 ng/ml IL-1β and/or 1 mM LNIO for six hours, where iNOS **(A)**, COX-2 **(B)**, aggrecan **(C)** and type II collagen **(D)**. Error bars represent the mean and SEM of eight replicates from three separate experiments, where (*) indicates comparisons between the different treatment groups and (ψ) indicates comparisons in unstrained constructs between untreated and IL-1β at 5 or 21% oxygen tension. All other comparisons were not significant (not indicated).

The effect of oxygen tension and FN-f on catabolic (iNOS and COX-2) and anabolic (aggrecan and type II collagen) gene expression is presented in Figure 5. Treatment with FN-f increased iNOS and COX-2 expression with a similar magnitude to IL-1β (all *P* <0.001; Figure 5A, B, respectively). In unstrained constructs, the effect of hypoxia increased iNOS, aggrecan and collagen type II (all *P* <0.001), but not COX-2 expression. At 5 and 21% oxygen tension, stimulation with dynamic compression alone resulted in a reduction of iNOS and COX-2 expression in constructs cultured with FN-f or IL-1β (all *P* <0.001).

Co-stimulation with compression and L-NIO marginally reduced iNOS but not COX-2 expression further. Conversely, fragment or cytokine treatment decreased aggrecan expression at 5 and 21% oxygen tension (both *P* <0.001; Figure 5C, D, respectively). In addition, the fragment, but not cytokine, reduced collagen type II expression at 21% oxygen tension (*P* <0.001; Figure 5D). At 5 and 21% oxygen tension, co-stimulation with compression and the NOS inhibitor restored aggrecan and collagen type II expression in fragment or cytokine treated constructs cultured at 5 and 21% oxygen tension.

### Compression inhibits FN-f induced cytokine production at 5 and 21% oxygen tension

**Figure 6:**
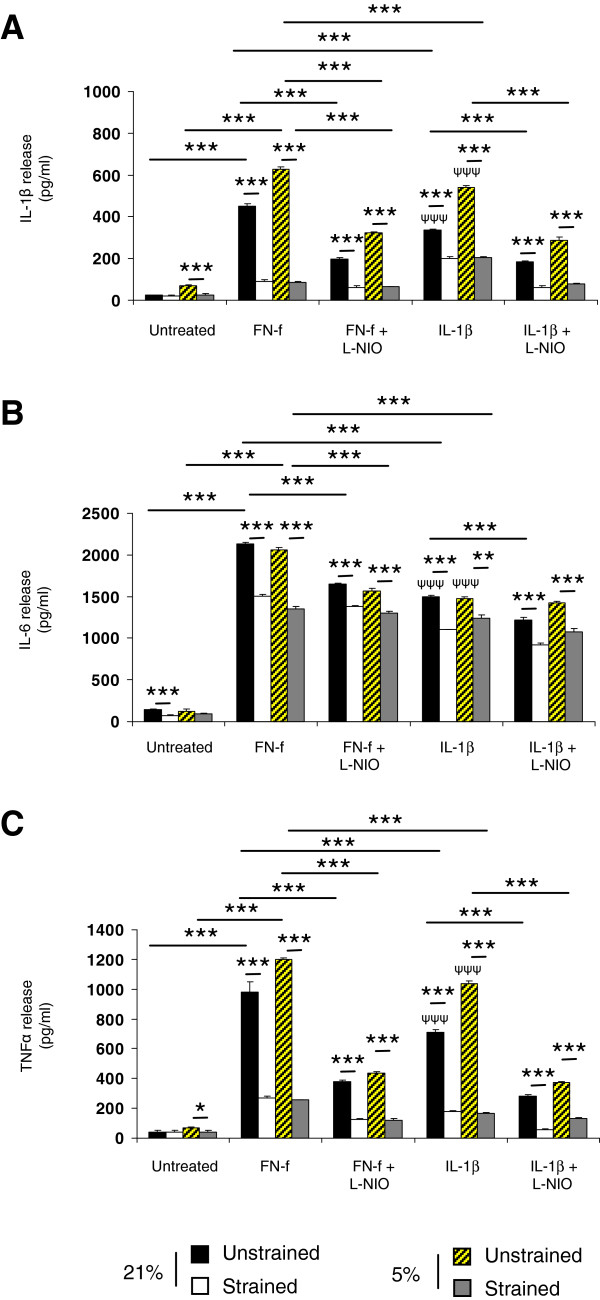
**Effect of oxygen tension, FN-f and dynamic compression on cytokine production. ** Chondrocyte/agarose constructs were cultured at 5 and 21% oxygen tension with 0 or 1 μM FN-f or 10 ng/ml IL-1β and/or 1 mM L-NIO for 48 hour where TNFα **(A)**, IL-1β **(B)** and IL-6 **(C)**. Error bars represent the mean and SEM of eight replicates from three separate experiments, where (*) indicates comparisons between the different treatment groups and (ψ) indicates comparisons in unstrained constructs between untreated and IL-1β at 5 or 21% oxygen tension. All other comparisons were not significant (not indicated).

We next examined the effect of FN-f, compression and oxygen tension on the production of pro-inflammatory cytokines and compared the response to constructs treated with exogenous IL-1β (Figure 6). In unstrained constructs, FN-f significantly increased IL-1β, IL-6 and TNFα production to a larger effect than the corresponding increase with exogenous cytokine treatment (all *P* <0.001). At 5% oxygen tension, the fragment or cytokine enhanced greater production of IL-1β and TNFα than at 21% oxygen tension. In contrast, the effects of fragment or cytokine on IL-6 production were broadly similar for constructs cultured at 5 and 21% oxygen tension. Cytokine production was reduced with dynamic compression and/or L-NIO in an oxygen-independent manner with the magnitude broadly similar for all treatments.

## Discussion

Matrix fragments are known to exert destructive effects in OA pathophysiology and regulate tissue remodelling events in chondrocytes [28]. In OA cartilage, exogenous cytokines increase iNOS expression and NO production leading to proteoglycan degradation [29,30]. The catabolic environment attempts to assist with tissue remodelling but the response will be influenced by mechanical factors. The question arises as to whether mechanical loading could act to slow down tissue damage during the early phase of the disease process or effectively contribute to OA progression. In addition, the levels of oxygen tension in the injured joint will have a significant impact on the metabolic processes thereby affecting the maintenance of fragment-induced pathway. The interactions among matrix fragments, oxygen tension and mechanical loading are, therefore, complex, and thus motivate the current investigation.

At 21% oxygen tension, FN-fs ranging from 29 to 140 kDa have been observed to bind to the pericellular matrix leading to NO, MMP and cytokine up-regulation, suppression of matrix synthesis and proteoglycan loss in a concentration-dependent manner [1,11,12,31-33]. We reported that the 29 kDa NH_2_-heparin binding fragment is highly active and increased NO and PGE_2_ production in a 3D/agarose model when compared with other matrix fragment types [16,25]. However, our previous studies tested commercially available fragments in contrast to the present work which generated FN-fs from cathepsin D and thrombin digests isolated from human plasma fibronectin, as previously described [24]. Our data are in agreement with previous findings, demonstrating a greater production of NO and PGE_2_ with FN-f when compared to IL-1β treatment and the response was reduced with NOS inhibitors. Interestingly, the fragment-induced catabolic response was greater at 5% when compared to 1 or 21% oxygen tension and resulted in an associated inhibition of matrix synthesis that was broadly similar at all oxygen tensions examined. However, we were unable to detect PGE_2_ synthesis in constructs cultured with fragment or cytokine under severe hypoxia (1%) conditions. Since prostaglandin synthesis involves the oxygenation of arachidonic acid into intermediates that are oxidised by COX leading to PGE_2_ synthesis, the oxygen concentration dependent effects will slow down production of inflammatory mediators. A similar response was reported in bovine chondrocytes treated with IL-1β in suspension culture, with a more pronounced increase of NO and PGE_2_ production at 5% when compared to 1 or 21% oxygen tension [34]. The effect of FN-fs and oxygen tension has not been previously investigated in chondrocytes. This is the first study to show that the fragment-induced catabolic effects are oxygen-sensitive and amplified under hypoxic conditions. Furthermore, studies on the effect of oxygen tension on the inflammatory response in chondrocytes have resulted in conflicting outcomes. Normoxia (20%) conditions were observed to produce greater levels of NO and PGE_2_ production in cytokine (IL-1α or TNFα) treated porcine explants when compared to severe hypoxia (1%) [35]. In contrast, moderate hypoxia (6%) reduced oxidative stress, stabilise hypoxia-inducible factor-1α (HIF-1α) expression and lowered MMP-9 levels in cytokine-induced chondrocytes compared to normoxia (21%) [36]. In OA chondrocytes, HIF-1α over-expression is known to be detrimental to cartilage physiology and its regulation by oxygen tension may present a potential therapeutic target for treating OA. In addition, the cytokine-induced production of NO at 5% oxygen tension was mediated by factors involved in the MAPK and NFκβ pathways, highlighting additional oxygen-sensitive mediators as potential targets for OA therapy [34,37,38]. Taken together, these studies emphasise the oxygen-dependency of the inflammatory response and suggest that further studies should examine the interplay of fragment and cytokine-induced pathways with oxygen tension.

**Figure 7:**
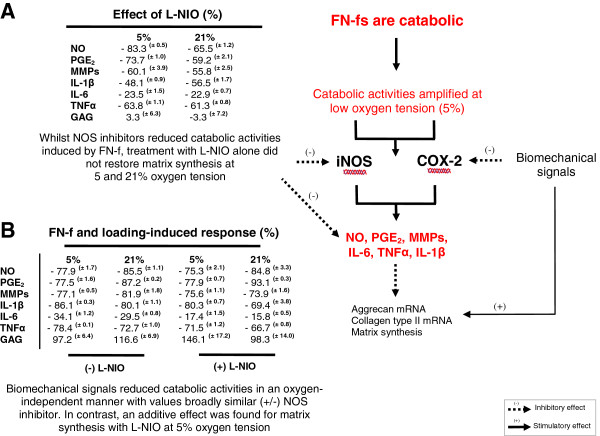
**Summary of interactions between FN-f, oxygen tension and biomechanical signals.** The presence of FN-f combined with low oxygen tension amplifies catabolic activities and increased production of NO, PGE2, MMPs and cytokines. Catabolic activities were reduced with L-NIO. However, the response was abolished following stimulation with biomechanical signals alone leading to restoration of anabolic effects. The magnitude in the FN-f and loading-induced response was broadly similar for FN-f treated constructs cultured in the presence and absence of L-NIO at 5 and 21% oxygen tension. Normalised values were expressed as a percentage change and either compared to unstrained constructs treated with FN-f and FN-f + L-NIO **(A)** or for unstrained and strained constructs treated with FN-f and FN-f + L-NIO **(B)**, where n = 8 to 12; ± SEM.

The effect of FN-fs and mechanical loading are summarised in Figure 7. Whilst NOS inhibitors reduced FN-f induced catabolic activities in unstrained constructs, treatment with L-NIO alone did not restore matrix synthesis (Figure 7A). In contrast, the loading-induced response was found to be oxygen-independent, such that stimulation with dynamic compression alone blocked fragment-induced catabolic effects at 5 or 21% oxygen tension (Figure 7B). Furthermore, the beneficial response associated with anabolic activities was supported by enhanced matrix synthesis and gene expression of aggrecan and collagen type II. This is the first report to show the combined effects of oxygen tension and dynamic compression on the suppression of fragment-induced catabolic events in an *ex vivo* model. It is well recognised that mechanical loading combined with oxygen tension will influence the production of inflammatory mediators. Indeed, in the absence of exogenous factors, mechanical compression increased NO production at 5 and 20% oxygen tension and the loading-induced catabolic response was reduced at 1% in porcine cartilage explants [21]. In contrast, long-term mechanical stimulation at 5% oxygen tension increased matrix synthesis and stabilised chondrogenic gene expression when compared to normoxic conditions (21%) in a chondrocyte/polyurethane model [39]. Conversely, cyclic stretch was reported to increase IL-8 and TNFα production in macrophages and the response was independent of oxygen tension [40]. However, it is not entirely known how biomechanical signals combined with oxygen tension influence the inflammatory pathways induced by fragments or cytokines. Indeed, at normoxic conditions, there is strong evidence which implicates the α5β1 integrin as a receptor for both mechanical loading and matrix fragments implicating overlapping pathways for these signals [41,42]. Integrin-mediated mechanotransduction will contribute to chondroprotective events resulting in an attempt by cells to stimulate anabolic processes locally and assist in tissue remodelling. However, conditions such as obesity or trauma that represent excessive or injurious loading will increase catabolic activities and accelerate matrix damage mediated by abnormal integrin signals [43,44]. Integrins serve as receptors for several matrix proteins involving the collagens and fibronectin. There may be shared ability among matrix fragments to disrupt integrin receptors at the cell surface and enhance receptor internalisation, thereby preventing integrin cluster formation and adhesion with matrix proteins. The disruption in normal interactions and changes in oxygen tension will influence matrix turnover, which, in turn, affects the ability of the tissue to respond to normal mechanical signals. It is not known whether oxygen tensions which exist in a diseased state (<5%) will aggravate mechanical and fragment-induced integrin signals and this should be explored further.

In free-swelling or unstrained constructs, the present study demonstrates for the first time that exogenous FN-f combined with low oxygen tension amplifies the production of catabolic mediators in an oxygen-dependent manner. In unstrained constructs, the catabolic effects were reduced with NOS inhibitors and abolished following stimulation with biomechanical signals alone. Both types of stimuli blocked the damaging effects induced by matrix fragments and restored anabolic activities in an oxygen-independent manner. Since FN-fs share the same receptors as mechanical loading, the effect of oxygen tension on integrin signals should be explored to identify whether integrin agonists or fragment oxygen-sensitive antagonists could be developed with *ex vivo* physio-biomimetic models. In addition, the mechanical environment clearly influences both reparative and tissue remodelling events *in vivo*, thereby emphasising the need to dissect the effects induced by fragments, oxygen tension and biomechanical signals. This knowledge could lead to the development of novel therapeutics for OA treatments.

## Conclusions

The 29 kDa NH_2_-FN-f is highly active and triggers the most damage *ex vivo*. The importance of fragment-induced damaging effects was highlighted in previous clinical studies which reported enhanced levels of FN-fs in human osteoarthritic cartilage and OA synovial fluids. Future therapeutics should, therefore, develop oxygen-sensitive molecular antagonists which are directed to specific domains of fibronectin. This could either prevent the generation of matrix fragments through antiprotease therapy or neutralise the effects of fragments by targeting multiple integrins. In addition, development of *ex vivo* physio-biomimetic models may help to examine the effect of an integrin agonist that maintains normal cell surface interactions with the extracellular matrix. Since physiological biomechanical signals are anti-inflammatory, this approach will facilitate development of therapeutics which slow down inflammation and allow the protective effects of moderate exercise or physiotherapy, to repair tissue and restore joint function *in vivo*.

## Abbreviations

DMEM: Dulbecco’s Modified Eagle’s Medium; FCS: Foetal calf serum; FN-f: Fibronectin fragment; HIF-1α: Hypoxia-inducible factor-1α; IL-1β: Interleukin-1β; iNOS: Inducible nitric oxide synthase; L-NIO: L-*N*-(1-iminoethyl)-ornithine; MAPK: Mitogen activated protein kinase; MMPs: Matrix metalloproteinases; NFκB: Nuclear factor-kappa B; NO: Nitric oxide; OA: Osteoarthritis; PGE2: Prostaglandin E_2_; TNF: Tumour necrosis factor.

## Competing interests

The authors declare they have no competing interests.

## Authors’ contributions

EP, TC and SV carried out the *ex vivo* experiments, performed biochemical, gene expression and statistical analysis, and drafted the manuscript. SV developed the MMP assay and interpreted the data. EP, TC, BPM and DB participated in the design of the study and analysed the data. TC conceived the study, and EP, DB, BPM, SV and WAWAB participated in its design and coordination and helped to draft the manuscript. All authors read and approved the final manuscript.
